# Colorectal Cancer associated with pediatric inflammatory bowel disease: a case series

**DOI:** 10.1186/s12887-021-02966-9

**Published:** 2021-11-11

**Authors:** Min Jee Kim, Jae Sung Ko, Minsoo Shin, Jong Woo Hahn, Soo Young Moon, Hyun Young Kim, Jin Soo Moon

**Affiliations:** 1 Division of Pediatric Gastroenterology, Hepatology and Nutrition, Department of Pediatrics, Seoul National University College of Medicine, Seoul National University Children’s Hospital, 101 Daehak-ro, Jongno-Gu, Seoul, 03080 Republic of Korea; 2 Department of Pediatric Surgery, Seoul National University College of Medicine, Seoul National University Children’s Hospital, Seoul, Republic of Korea

**Keywords:** Pediatric-onset inflammatory bowel disease, Colorectal cancer, Crohn’s disease, Ulcerative colitis, Surveillance endoscopy

## Abstract

**Background:**

Inflammatory bowel disease (IBD) is associated with an increased risk of Colorectal cancer (CRC), and its most important risk factors are the duration and extent of the disease. Pediatric-onset inflammatory bowel disease has a tendency for a more extensive, more severe, and longer predicted disease duration than adult-onset inflammatory bowel disease. This study aimed to identify the clinical characteristics of patients with CRC related to pediatric-onset IBD and consider the appropriateness of current surveillance endoscopy recommendations for the detection of premalignant lesions and early-stage CRC.

**Methods:**

We searched a research platform based on the SUPREME electronic medical record data-mining system to identify cases of colorectal malignancy in patients with pediatric IBD that presented between 2000 and 2020.

**Results:**

During the follow-up, 4 (1.29 per 1000 person years) out of 443 patients with PIBD was diagnosed with CRC. The median age at diagnosis of CRC was 18.5 (range: 15–24) years, and the median period from diagnosis of IBD to CRC was 9.42 (range: 0.44–11.96) years. The sigmoid colon was the most frequent location of CRC (in 3 of the 4 cases). Adenocarcinoma was the most common histological type (in 2 of the 4 cases).

**Conclusions:**

Patients with pediatric-onset IBD exhibited a much shorter disease duration than that of adult-onset IBD at the time of diagnosis of CRC, suggesting that surveillance endoscopy for the detection of precancerous lesions and early-stage cancer should be initiated earlier in pediatric patients than in adult patients.

## Background

Inflammatory bowel disease (IBD) is associated with an increased risk of colorectal cancer (CRC). The most important and well-recognized risk factors for CRC in patients with IBD are the duration and extent of the disease [1, 2]. The presence of primary sclerosing cholangitis, inflammatory complications, and a family history of CRC are also established risk factors [3, 4]. Pediatric-onset IBD (PIBD) is associated with a tendency for a more extensive and more severe disease with a longer anticipated duration, as well as a higher need for immune suppression earlier in the disease course, than adult-onset IBD [5–7]. Previous reports have found the increased risk of cancers in PIBD [8, 9] and a nation-wide study in Sweden suggested 18-fold increase in CRC in PIBD [9]. The risk of CRC is one of the major concerns when managing patients with PIBD, but limited data is available about the clinical characteristics of patients with PIBD who develop CRC. In addition, there is no evidence based guideline for surveillance endoscopy in PIBD. This study aimed to identify the clinical characteristics of patients diagnosed with CRC following a diagnosis of PIBD at a single center over the past 20 years and to consider the appropriateness of current surveillance endoscopy recommendations for the detection of premalignant lesions (dysplasia) and early-stage asymptomatic CRC.

## Methods

We reviewed the clinical data of patients diagnosed with CRC after being diagnosed with PIBD between 2000 and 2020 at Seoul National University Children’s Hospital in Korea, and the study was approved by the Institutional Review Board of Seoul National University Hospital (IRB No. 2009–093-1157) with a waiver of informed consent.

A research platform was utilized based on an electric medical record data-mining system (“SUPREME”) to search for patients. Multiple sets of key words including disease name (“Crohn’s disease”, “ulcerative colitis”,), 10th revision of the International Statistical Classification of Diseases and Related Health Problems (ICD-10) codes for CRC (“C18-C21”), diagnosis date (“from 2000 to 2020”), diagnosis age (“from 0 to 17 years old at diagnosis”) and internal code for identifying Rare Intractable Disease (RID) registration (“2002599, 2002608, 187, 195, 227, 228, 317, 318, 344, 346-348, 354-356, 358, 359”) were used.

In our institution, diagnoses of pediatric CD and UC were made in accordance with published guidelines [10, 11] and all patients with IBD were registered in the RID database managed by the National Health Insurance [12]. Retrieved data included age at first IBD diagnosis, IBD type, genetic findings if present, disease location, and presence of complications around the anus or luminal complications. Information regarding the drugs used before cancer diagnosis was also collected. The diagnosis of cancer was confirmed through histological findings, and information on the age at cancer diagnosis, histological type, stage, and location of CRC was retrospectively collected from the electronic medical records. Continuous variables such as age and years were described as medians with ranges. We calculated crude rates by dividing the number of CRC diagnosed during follow up by the corresponding person time at risk.

## Results

A total of 443 pediatric patients (aged < 18 years) were diagnosed with IBD between 2000 and 2020 at Seoul National University Children’s Hospital. The end of follow-up was the date of their last outpatient visit. The follow-up period for each patient varied significantly, from under 1 year to 20 years, and 199 (44.9%) patients had more than 7 years of follow-up. In 3102 person years of follow-up, we found 4 CRC in patients with PIBD (1.29 per 1000 person years).

The characteristics and details of the four patients are shown in Table 1. All patients were male and the median age at diagnosis of IBD was 11.5 (range: 5–16) years. The underlying IBD type was CD in two patients, UC in one patient and very early-onset IBD (VEOIBD) in one patient. Most patients had an extensive range of disease invasions on colonoscopy, and patient 4 was only able to undergo an examination of the sigmoid colon because of luminal narrowing. Two patients had perianal complications, and two patients had luminal complications, such as bowel obstruction. All patients were treated with thiopurine for a median dosing period of 2.27 (range: 2.05–9.43) years. Patient 2 was also administered a tumor necrosis factor inhibitor for 3.44 years, methotrexate for 0.33 years, and golimumab for 0.71 years. None of them had ever undergone surgery before the diagnosis of cancer. The median age at the diagnosis of CRC was 18.5 (range: 15–24) years. The median period from the diagnosis of IBD to CRC was 9.42 (range: 0. 44–11.96) years. Patient 4 was diagnosed with cancer almost immediately after being diagnosed with IBD, and the cancer had already progressed to the extent that peritoneal metastasis was confirmed with ascites, suggesting a case of delayed IBD diagnosis. Therefore, when this patient was excluded, the median period was 9.93 (range: 8.09–11.96) years. CRC was located in the sigmoid colon in three patients (including one patient with cancer in both the sigmoid and ascending colon) and transverse colon in one patient. Patient 1 and 2 underwent total colectomy with ileorectal anastomosis. Patient 3 and patient 4 underwent total proctocolectomy with ileal pouch-anal anastomosis and subtotal colectomy with ileostomy respectively. Three patients (1, 2 and 3) were diagnosed with early stage and the other with terminal stage of colon cancer. Adenocarcinoma was the most common histological type (2 of 4), and the remaining cases were mucinous carcinoma in one patient and signet ring carcinoma in one patient. Patient 4 took a palliative chemotherapy after surgery but died from aggravation of end-stage cancer. The others with early stage colon cancer continued to undergo follow-up in the outpatient clinic without chemotherapy.

**Table 1:** Clinical details at diagnosis and during follow-up in patients with PIBD-related CRC

Patient No.	Patient 1	Patient 2	Patient 3	Patient 4
Sex	M	M	M	M
Age at first IBD diagnosis (years)	12	11	5	16
Year of first diagnosis	2000	2010	2001	2018
Type of IBD	CD	UC	VEOIBD	CD
Genetic findings	NA	NA	Negative	NA
Disease location (Paris classification)	L3	E4	L3	L4
Perianal diseases	Fistula	Skin tag	(−)	(−)
Luminal complications	Luminal stricture Bowel obstruction	(−)	(−)	Luminal narrowing Bowel obstruction
Thiopurine (years)	9.43	2.05	2.48	0.42
TNF inhibitor (years)	(−)	3.44	(−)	(−)
Methotrexate (years)	(−)	0.33	(−)	(−)
Other biologicals (years)	(−)	0.71 (Golimumab)	(−)	(−)
Surgery before cancer diagnosis	(−)	(−)	(−)	(−)
Age at cancer diagnosis (years)	24	20	15	17
Location	Transverse colon	Sigmoid colon	Ascending, sigmoid colon	Sigmoid colon
Histological type	Mucinous carcinoma	Adenocarcinoma	Adenocarcinoma	Signet ring carcinoma
Stage according to AJCC 8th	pT3N0M0	pT1N0M0	pT3N0M0	pT4aN2bM1
Disease duration before cancer diagnosis (years)	11.96	8.9	9.93	0.44
Last follow-up (year)	2020 (Ongoing)	2020 (Ongoing)	2020 (Ongoing)	2019 (Expired)

*IBD* inflammatory bowel disease, *TNF* tumor necrosis factor, *AJCC* American Joint Committee on Cancer, *NA* not applicable

## Discussion

We analyzed four cases of PIBD-related CRC at a single center in Korea. The case of VEOIBD (patient 3) which the initial diagnosis was UC but revised to CD immediately after the diagnosis of cancer was previously published [13]. A subset of patients with VEOIBD is known to present with extensive colitis, which can make it difficult to differentiate UC from CD. According to a previous study, 25% of the children with VEOIBD were originally diagnosed with UC or unclassified IBD, but the diagnosis was changed to CD over time [14, 15]. Patient 3 underwent genetic testing to identify monogenic mutations associated with VEOIBD, but the result was negative.

In our study, 4 (1.29 per 1000 person years) patients was diagnosed with PIBD-related CRC during follow up. However in nationwide cohort study in Sweden, among 9405 patients with PIBD, 122 (0.8 per 1000 person years) people with PIBD had CRC with hazard ratio of 33 for UC and 5.8 for CD [9].

All patients except one had a disease duration of at least 8 years at the time of diagnosis of CRC. The median disease duration was 9.42 (range: 0.44–11.96) years (if patient 4 was excluded, 9.93 [range: 8.9–11.96] years), which is much shorter than expected for adult patients with IBD [16, 17]. The mean duration of adult onset CD until the diagnosis of CRC was 18.3 years in a meta-analysis of English and German articles [16], and the overall duration of adult onset UC in patients with UC-associated CRC was a median 19 years [17].

A case-control study revealed that the risk of colorectal neoplasia in patients with UC exhibited a highly significant correlation with the degree of colonoscopic and histological inflammation [18]. In the present study, all patients exhibited widespread disease invasion on colonoscopy except for one, in whom disease invasion was difficult to evaluate because of luminal narrowing. All four patients commonly used thiopurine for a median dosing period of 2.27 years. The relationship between cancer and thiopurine in IBD is complex because of its both anti-inflammatory effect and carcinogenic effects [19]. Thiopurine is known to be associated with an increased risk of several types of cancer including non-melanoma skin cancer, lymphoproliferative/myeloid disorders, urinary tract cancer and cervical high-grade dysplasia/cancer [19]. According to a meta-analysis, there is no significant protective effect of treatment with thiopurines on the risk of colorectal neoplasia in patient with IBD [20].

Children with IBD are anticipated to have a longer disease duration than adults, and numerous studies have reported that PIBD tends to be more severe and extensive than adult-onset IBD [5–7]. In our study, patients with PIBD-related CRC tended to develop cancer earlier in the disease period than adults, which suggests that the risk of CRC is one of the major concerns when managing patients with PIBD. Previous studies suggested that appropriate surveillance endoscopy could lower the incidence of CRC or detect CRC in earlier stages, thus leading to improvements in prognosis, although lead time bias might substantially contribute to this benefit [21, 22]. Guidelines for adult IBD suggest that surveillance colonoscopy should be initiated for dysplasia between 6 and 10 years after diagnosis. Currently, the widely adopted time point is 8 years of disease because the risk of CRC is significant after this point and increases in subsequent years [22–27]. Guidelines from the British Society of Gastroenterology suggest a surveillance interval of 3 or 5 years depending on the risk factors, while guidelines from the American Gastroenterological Association recommend intervals of 1–3 years [24, 25]. Despite current surveillance strategies, a Scandinavian population-based study suggested that patients with UC exposed to increased risk of CRC diagnosis and CRC death, indicating that current surveillance programs still seems to be room for improvement [4].

As the risk of CRC in patients with PIBD is as high as that in adults, while the disease duration until the diagnosis of CRC tends to be shorter than that in adults, surveillance endoscopy should be initiated earlier than currently recommended for IBD. Further studies are required to determine the appropriate time to begin surveillance and the optimal surveillance interval.

As this study had a retrospective design, we relied on electronic medical records to obtain all patient information. The main limitation of this study is that the number of patients with CRC was small, which made it unclear whether the median values obtained were representative of actual patients with PIBD who developed CRC. The inclusion of additional cases will lead to more convincing results. Furthermore, the small number of patients prevented us from performing subgroup analysis based on a diagnosis of CD or UC, and all patients were classified into a single IBD group. The lack of a control group also made it impossible to elucidate the extent to which CRC cases were linked to IBD.

## Conclusion

In our study, disease duration of PIBD until the diagnosis of CRC was shorter than that of adult-onset IBD. Thus, surveillance endoscopy for precancerous lesions (dysplasia) and early-stage cancer should be initiated earlier in patients with PIBD than in patients with adult-onset IBD. Further studies are required to determine the appropriate time to begin surveillance and the optimal surveillance interval.

## Data Availability

The database used and/or analyzed during the current study are available from the corresponding author on reasonable request.

## Abbreviations

IBD: Inflammatory bowel disease
CRC: Colorectal cancer
PIBD: Pediatric-onset inflammatory bowel disease
CD: Crohn’s disease
UC: Ulcerative colitis
RID: Rare intractable disease
VEOIBD: Very early-onset inflammatory bowel disease
